# The Effects of Anastomotic Leaks on the Net Revenue from Colon Surgery

**DOI:** 10.3390/ijerph19159426

**Published:** 2022-08-01

**Authors:** Bassey Enodien, Andreas Maurer, Vincent Ochs, Marta Bachmann, Maike Gripp, Daniel M. Frey, Anas Taha

**Affiliations:** 1 Department of Surgery, GZO Hospital, 8620 Wetzikon, Switzerland; bassey.enodien@gzo.ch (B.E.); marta.bachmann@gzo.ch (M.B.); maike.gripp@gzo.ch (M.G.); daniel.frey@gzo.ch (D.M.F.); 2 Department of General Surgery, Hospital Rheinfelden, 4310 Rheinfelden, Switzerland; andreas.maurer@gzf.ch; 3 Department of Business and Economics, University of Basel, 4052 Basel, Switzerland; vincent.ochs@unibas.ch; 4 Department of Biomedical Engineering, Faculty of Medicine, University of Basel, 4123 Allschwil, Switzerland

**Keywords:** anastomotic leakage, net revenue, colon surgery

## Abstract

Background: Complications in colon surgery can have severe health consequences, while at the same time, they are associated with increased costs. An anastomotic leak (AL) is associated with significantly increased costs compared to cases without. The aim of our analysis was to evaluate, which individual processes and patient-unrelated factors influencing the treatment process of colon surgery are responsible for the financial burden in patients with AL. Methods: Data from 263 patients who underwent colon surgery in Wetzikon hospital between January 2018 and December 2020 and was analyzed. In these 263 cases, 12 anastomotic leaks occurred and were compared with 36 cases without AL using a Propensity Score Matching (PSM). The covariates for the PSM have been Age, Sex, and Type of Surgery (t value: −3.26, *p*-value: 0.001). Results: A total of 48 surgeries were broken down in terms of costs and profitability. This reflected a mean deficit of −37,527 CHF per case (range from −130.05 to +755 CHF) for patients with AL, whereas a mean profit of 1590 CHF per case (range from −24.37 to +12.65 CHF) for those without AL (*p* < 0.001). Thus, the difference in profit showed a factor of 24.6 with an overall significant negative outcome for the occurrence of AL. The main cost contributing factors were the length of hospital stay (~*p* < 0.05) and length of intensive care (*p* < 0.05), whereas neither surgical operation time and anesthesia time nor surgical access, insurance status, indication or type of operation had a significant influence on the net revenue. Conclusion: AL after colon surgery leads to a significant deficit regarding the net revenue. Regarding process optimization, our analysis identified several sectors of non-patient-related, yet cost-influencing variables that should be addressed in future evaluations and optimization of the colon surgery treatment processes.

## 1. Introduction

Anastomotic leakage (AL) is one of the severe complications after colorectal surgery with an AL rate that has been reported between 1% and 28% [1,2,3,4,5]. AL is not only linked to increased morbidity, mortality, and poor oncological outcome [4,5,6], but also to increased costs [7]. In addition, insufficient treatment processes can lead to reduced quality of life and suffering for the patient. Translated into business economics, one can speak of the limited quality of the treatment process and increased costs.

A 2020 Cochrane review identified surgeon experience, anastomosis technique, and protective stomas in low anterior resection as significant factors influencing AL rates [5]. In contrast to these studies investigating the risk factors for anastomotic leakage, from a managerial perspective, AL in colorectal surgery has a relevant negative impact on the financial outcome of the SwissDRG system and the quality of the treatment process [7,8,9,10]. It is postulated that there are factors in the treatment process of colon surgery that are responsible for the financial loss and can be mapped. Therefore, our analysis has included individual processes and factors in the operating room, the nursing departments, and the administration to define which relevant influencing factors in the treatment process of colon surgery are responsible for the negative outcome in patients with AL concerning quality and costs. This knowledge is essential to optimize the treatment process within the framework of process management. The goal of this process optimization is to increase quality while reducing costs.

There are no studies in the literature on process optimization in the context of AL with convincing results. However, this knowledge is crucial to improving the financial outcome by reducing costs and at the same time increasing quality, according to the “lean philosophy” by Angerer [11]. In the healthcare system or “lean healthcare”, the philosophy of process management was coined in the 1950s by the Japanese economist Taiichi Ohno [12]. In this context, AL and its frequency as a complication of colonic resection fall into financial and performance indicators [11].

Our analysis evaluates, which individual processes and patient-unrelated factors influencing the treatment process of colon surgery are responsible for the financial burden in patients with AL.

## 2. Materials and Methods

### 2.1. Data Collection

Data from all the patients admitted to Wetzikon hospital between January 2017 and December 2020 who underwent colon surgery were included in this study (*n* = 263). The Wetzikon hospital is a private hospital with a public service contract. All patients with AL (*n* = 12) were matched 1:3 to those best suitable without AL (*n* = 36) out of the remaining patients (*n* = 251); matching criteria were gender, age, and type of surgery. For the matching method, logistic regression has been used to run a PSM (t value: −3.26, *p*-value: 0.001). Of these 12 patients with AL, 4 patients underwent hemicolectomy left or extended hemicolectomy left, 4 hemicolectomy right or extended hemicolectomy right and 4 patients underwent sigmoidectomy. These patients were matched as described with 36 patients without AL. In total, 16 patients underwent hemicolectomy left or extended hemicolectomy left, 16 hemicolectomy right or extended hemicolectomy right and 16 Patients underwent sigmoidectomy.

### 2.2. Variables and Definitions

Data on age, sex, diagnosis, surgical operating time, anesthesia time, real anesthesia time, hospitalization, insurance status, intensive care time, surgery approach, and financial costs and revenues were collected. The insurance status was either compulsory basic care or semi-private and private hospital care. Differences are the coverage of extra services, which means the patient could choose the hospital physician and is entitled to a single bedroom (private) or double bedroom (semi-private). Private and semi-private are summarized in the following. Finally, data on whether a patient had anastomotic leakage or not during the operation were added.

Anastomotic leak (AL) was defined as a leak of luminal contents or gas from the surgical joint between the hollow viscera. Luminal contents emerged either through the wound or at the drain site, or they were collected near the anastomosis. All AL were verified by CT-Scan with contrast media. Patients showed signs of clinical deterioration and/or abnormal laboratory findings before CT-Scans were initialized. Routine laboratory testing was performed on the 2nd and 4th postoperative days.

There were no strict ERAS protocols used in the study population.

Because Wetzikon hospital is in Switzerland net revenue is shown in swiss franc (CHF) the exchange Ratio between Euro (EUR) and Swiss franc (CHF) is 1:1. The given swiss franc (CHF) values equal the same values in Euro (EUR).

A structured overview of the variables relevant to this analysis is presented in Table 1 In this table, all the variables are described based on their definition. Additionally, the formula for how the variables and their values were calculated is shown.

### 2.3. Statistical Analysis

With no missing data in the dataset, Welch’s two-sample *t*-test was conducted to compare the characteristics of patients with anastomotic leaks (AL = “yes”) and those without (AL = “no”). All continuous variables are reported as means with their standard deviations (SD), while categorical variables are treated as numbers with their percentages.

In addition to that, the continuous variables have been reported as medians and their respective interquartile differences in Table 2.

The effects of the relevant variables on the net revenue between the two groups were determined based on linear regression and an Anova analysis. For the statistical analysis, the free software “R Version 4.0.5” (R Core Team, Vienna, Austria) of the R Project for statistical computing from the R Foundation was used [13]. The significance level for the entire analysis was defined as *p* < 0.05.

## 3. Results

Between 2017 and 2020, 263 patients were operated on for various indications with colonic resection and colonic anastomoses. Twelve anastomotic leakage cases occurred (5%).

These twelve cases were matched with 36 patients without an anastomotic leakage. The median age of the 48 selected patients was 76 years (52–94 years, IQR: 10); 31% of the patients were women, 69% of the patients were men, 26 laparoscopic and 22 open surgery approaches were performed. The median operating time was 166.5 min (39–571 min, IQR: 84.75 min), and the median anesthesia time was 93 min (45–467 min, IQR: 70.5). Among the patients, 39 patients had general insurance, whereas six patients had semi-private insurance, and three patients had private coverage. The main indication for surgery was tumor surgery (73%). The median length of hospital stay was 5.5 days (1–84 days, IQR: 13.5). While patients without AL stayed 0 (0) days in the hospital, those with AL had a prolonged hospital stay of 32.5 (IQR: 18.75) days. The median of the intensive care stay was 0 days (0–70 days, IQR: 1) in total, here patients without AL stayed 2 (0.64, IQR: 2.75) days on average, while those suffering from AL as a postoperative complication stayed 2 (19.69, IQR: 2.75) days in an intensive care unit (see Table 2).

The 48 surgeries were additionally broken down in terms of costs and profitability. There was a mean deficit of −37 CHF per case (ranging from −130 to +755 CHF) for patients with AL, whereas a mean profit of 1590 CHF per case (ranging from −24.37 to +12.65 CHF) for those without AL. The occurrence of an anastomotic leak led to a significant loss in net revenue (*p* < 0.01).

**Figure 1:**
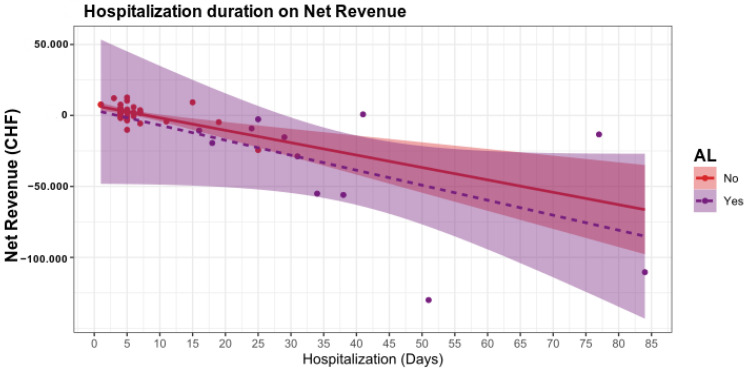
The length of hospital stay is a significant factor influencing the net revenue. In patients without AL, the duration of hospitalization has a minor influence on the net revenue, whereas in patients with AL, losses are already expected from day 4 of hospitalization.

**Figure 2:**
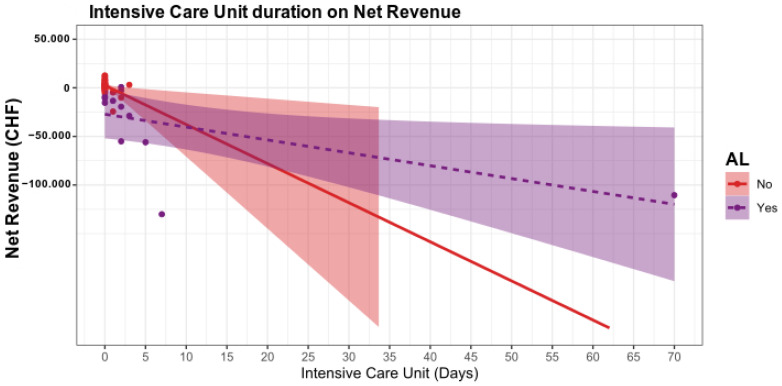
The influence of the intensive care unit stay is significant. In patients without AL, the duration of intensive care stay only has a minor influence on the net revenue. Whereas in patients with AL, losses are expected from the first day of the intensive care unit stay.

In a regression model, we evaluated further factors influencing the net revenue. Here, the length of hospital stay (~*p* < 0.05, Table 3 and Figure 1) and the number of days of intensive care (*p* < 0.05, Table 3 and Figure 2) significantly decreased the net revenue.

**Figure 3:**
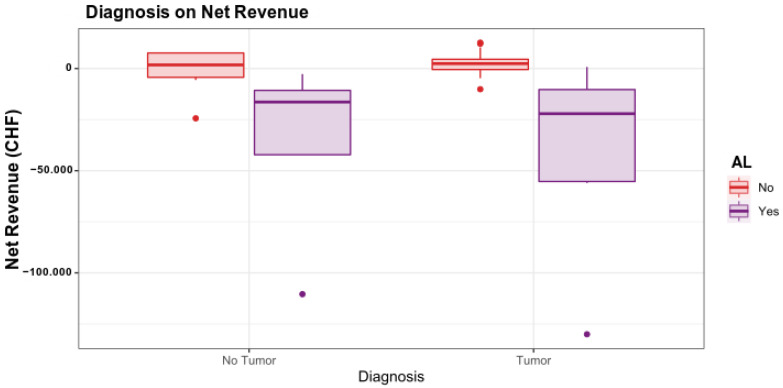
The influence of the diagnosis on the result differs markedly in the two populations of patients without and those with AL. For patients without AL, the type of diagnosis only has a minor influence on the net revenue. 1, No Tumor; 2, Tumor.

The influence of the single indications on the net revenue is demonstrated in (Figure 3). In the regression model, when displaying the diagnoses in detail, tumor surgery tends to have a more considerable loss of net revenue if an AL occurs than the other indications.

In addition, the operation did not significantly contribute to a loss in net revenue (Table 3).

**Figure 4:**
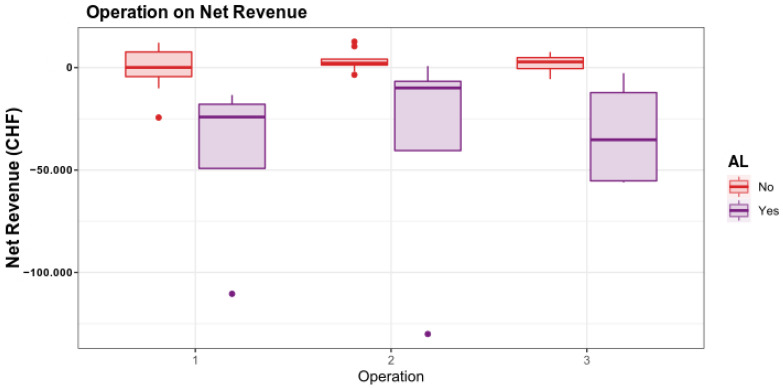
The influence of the operation on the result differs markedly in the two populations of patients without and those with AL. For patients without AL, almost every type of operation shows stable higher net revenue. In cases with AL, mainly hemicolectomy left, extended left and sigmoidectomy led to a high financial loss. 1, hemicolectomy left or extended hemicolectomy left; 2, hemicolectomy right or extended hemicolectomy right; 3, sigmoidectomy.

Nevertheless, when breaking down the operations, there is a trend of higher financial loss for patients undergoing hemicolectomy left or extended left, if an AL occurs in the following course (Figure 4).

**Figure 5:**
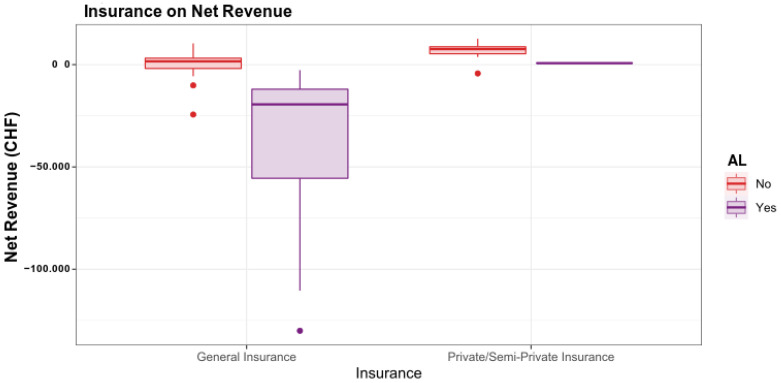
The type of health insurance policy (general insurance vs. semi-private/private insurance) determines the impact of an AL on the net revenue. If no AL occurs, the type of insurance has a minor influence on the net revenue compared to patients with AL. If an AL occurs, losses are expected mainly for general insured patients.

The insurance status did not significantly influence the net revenue in the regression model (*p* = 0.143). Thus, all patients suffering from AL with general insurance ended up with a negative net revenue (Figure 5).

## 4. Discussion

Anastomotic leakage after colon surgery is a significant postoperative complication linked to increased morbidity and mortality and a tremendous impact on health care costs. Our analysis sought to investigate the economic impact of AL in a hospital remunerated by the SwissDRG-based system and to identify which individual processes and patient-unrelated factors influence the treatment process.

The overall AL rate in our population of 263 patients was 5%, in agreement with previous literature [10,14,15,16]. Our analysis confirmed a significant financial loss for patients with AL after colon surgery with a mean loss of −37.52 CHF per case, while a mean gain of 1590 CHF per case for patients without AL. This result equals 24.6 times the cost of a colonic resection without an AL, corresponding quite well to the calculation by La Regina et al. who found a financial impact of AL in colorectal surgery with 23.4 times decreased reimbursement [7]. Hammond et al. found in their retrospective analysis of 99.879 patients using the US Premier Perspective™ database a total of 6.18% of patients with AL, of which had significantly higher mean costs of $72.90 compared to $25,005 for non-AL patients. While investigating the influence of AL on the whole intestinal tract, Turrentine et al. found similar results, 3.5-fold higher hospital costs for cases with AL in a retrospective collection of 2237 cases at the University of Virginia between 2003 and 2006 [10]. Ashraf et al. investigated the impact of AL regarding the remuneration after low anterior resection in the English national healthcare system (NHS). They found 2.7 times higher costs for patients with AL than their counterparts without this complication after elective resections in 23.388 patients [17]. Although all the mentioned studies confirmed significantly higher costs associated with AL in colonic and gastrointestinal surgery, generalization and transferability of economic implications among different countries and healthcare systems are often elusive, and comparisons require consideration of the complexities of local reimbursement systems.

We demonstrated in our cohort that the critical treatment processes causing the high costs are the stay in the intensive care unit (2.1 days without AL vs. 7.83 days with AL) and the length of hospital stay (14.4 days without AL vs. 39 days with AL). These factors are expected to be the direct effect of AL as a severe health complication of colon surgery and go along with the observation of La Regina et al. They identified length of hospital stay as the most cost-producing factor in eight patients after rectal resection suffering from AL [9]. Similarly, in the large-scale analysis by Hammond et al., length of hospital stay was found to be a significant contributing factor to the increased costs [7]. Likewise, Agzarian et al. investigated the costs of resource consumption for treating 24-grade III–IV anastomotic leaks after esophageal resections. The prolonged intensive care unit stay was the largest contributor with 30% of costs in their analysis [18]. Although the severity of a diagnosis is recorded for remuneration in the case-based lump sum rate system SwissDRG as part of the data entered in the grouper software [19], these are not sufficiently considered in the level of remuneration [20]. Therefore, prolonged hospitalizations and stays in the intensive care unit for patients with severe complications such as AL lead to a financial deficit for the hospitals [20]. This effect is not accidental or unintentional, since the introduction of SwissDRG in 2012 was intended, among other things, to build up cost pressure aiming to increase quality and optimize processes [19]. Under the current framework conditions, it is therefore in the best interest of hospitals to increase the quality of their treatments to limit such costly complications.

In our analysis, the surgical approach (laparoscopic vs. open), indication, and operation type did not significantly influence the net revenue. However, tumor patients with AL had a trend to show higher financial losses compared to other diagnoses. Likewise, the study by La Regina et al. demonstrated no relevant difference in financial outcome for surgical technique (laparoscopic vs. open) but no difference in the presence of carcinoma [7].

Another sub-analysis has been conducted to check whether the costs of mechanical sutures are significantly higher than the manual ones. In order to do so, we have focused on the AL cases and ran a regression on that. The results show that the Stapler cases for AL have a small significant negative impact on the final revenue (Table 3).

For the Rahbari Score, our results indicate that there are no significant statistical differences for the AL cases, meaning that the cost per case did not differ greatly due to the Rahbari Score.

Unlike findings in the literature, our analysis showed no significance in net revenue regarding age [8]. This factor causes costs but cannot be influenced and, consequently, is not relevant for revenue enhancement and analysis, which aims to improve process management. In the Swiss public healthcare system, public service contracts are issued, and hospitals are obligated to care for all patients, therefore a preselection of patients based on age would not be feasible.

Strategic management and process management fields are uncharted territory for many surgeons because their training has been characterized by decades of learning clinical skills in addition to clinical and experimental research. Regarding business concepts such as process management, the healthcare system lags 10–20 years behind the industry in terms of development. The increasing financial pressure on hospitals, growing with the introduction of the SwissDRG in 2012 [19], is steadily increasing with the lowering of the base rates in the SwissDRG system. Many hospitals will no longer be able to operate profitably without new concepts [21,22]. New strategies and other measures (purchasing management, new construction projects, and mergers/collaborations) for increasing profits and optimizing processes must be developed to remain competitive in the future [21]. The efficiency achieved through process management leads to decreasing costs and increasing quality [11]. Focusing on process optimization, the prevention and early treatment of AL plays a key role. The first approach of process management with optimization in colon surgery that has gained worldwide acceptance has been enhanced recovery after surgery (ERAS) programs [23]. These ERAS programs led to process optimization by shortening hospital stays by 30% to 50% [23]. Approaches for prehabilitation with the aim of reducing postoperative complications are currently being reexamined [24], after previously published studies have not shown clear and satisfactory results [25,26].

Furthermore, treatment pathways in surgical clinics have increased patient satisfaction. Here, the impact was measured by patient-reported outcome measures (PROM) and showed an increase in patient satisfaction [27]. In a second step, this feedback should be used for process analysis and process optimization to improve the quality of care. A future focus of research must be on concepts for process optimization and the management of AL in colon surgery, as this is one of the most significant complications in the treatment process from a patient’s point of view and a business perspective.

Nonetheless, our analysis has several limitations. The main limitation of our study is the small number of cases, and all shortcomings of that caveat apply. With only 12 patients in the AL group, the limited sample size: allows only for limited analysis and limited reliability of results. We did not perform subgroup analyses and potentially significant results could be masked. Yet, the data display the results of colon surgery of a four years period in a mid-volume Swiss hospital and several comparisons reached statistical significance.

Furthermore, the information gathered during our study was collected from a single Swiss hospital. However, we expect that our results can be used to predict the cost calculations and repayments related to complexities at other Swiss institutions. Furthermore, since other nations adopted repayment systems based on a common DRG idea, our results may even reflect the revenue system of other countries, considering the differences in DRG-based installments.

More limitations of this study are that data on prior operations was missing, obesity and other known confounders for AL, and prolonged hospital stays were not included in this analysis. Another limitation of this study is that other operations could have had an impact on the net revenue.

Regarding process optimization, we did not focus on single processes on the ward or the intensive care unit. Nevertheless, we identified cost-influencing areas in colon surgery that can be addressed for revenue enhancement and need to be closer analyzed concerning process optimization. Such as that should be refined in future analysis to ameliorate particular performances effectively.

## 5. Conclusions

Besides the severe health consequences for individuals, cases of AL have a significant cost burden in the SwissDRG system. Enhanced costs are primarily for resource utilization of intensive care costs and prolonged hospital stays. Complications like AL represent a loss of quality in the treatment process in colorectal surgery. In addition to efforts toward robust prevention of AL, further optimization processes in patients should focus on these cost-influencing variables and need to be broken down in more detail to minimize the significant financial and quality impact, not to mention the health consequences for affected patients.

## Figures and Tables

**Table 1:** Variables used for statistical testing with their definition and a formula for the parameters.

Variable	Definition	Formula/Categories
Age	Patients age in years	Date of operation—patient‘s date of birth
Sex	Gender	Male/female
Diagnosis	Type of diagnosis	Tumor/Others, No Tumor
Surgical operating time	Period from first surgical cut to final surgical suture (in minutes)	Timepoint (final suture)—timepoint (first cut)
Anesthesia time	sum of induction time and emergence Time as reported by the anesthesiologist (in minutes)	Induction time + emergence time
Hospitalization	Days at the hospital (in days)	
Insurance status	Coverage status of patient‘s procedure by one of the healthcare providers	General Health insurance or semi-private/private health insurance
Intensive care time	Intensive care time of a patient (in days)	
Surgical approach	Surgical approach	Open/laparoscopic
Net revenue	Net revenue of a case	Revenue–final costs
Revenue	Amount of CHF which the hospital earned	
Final costs	Sum of all types of costs	Direct costs + care costs + administrative costs + infrastructure costs
Rahbari Score	Anastomotic Leakage grading system	Level A: AL results in no change, Level B: AL requires intervention but no relaparatomy, Level C: AL requires relaparatomy
Anastomotic Technique	Techniques for the surgical approach	Hand-Sewn, or Stapler

**Table 2:** Descriptive statistics of the sample.

Variable	Overall	AL	No AL	*p* Values
**Patients (*n*)**	48	12	36	N/A
**Age (median/IQR)**	76 (10)	76.5 (9.5)	75.5 (11)	0.768
**Sex male (*n*)**	26 (54.1)	6 (50)	18 (50)	0.48
**Surgery approach (laparoscopic) (*n*/%)**	26 (54.16)	3 (2.5)	23 (63.8)	N/A
**Hospitalization days (median/IQR)**	5.5 (13.5)	32.5 (18.7)	5 (2)	0.001 *
**Intensive care days (median/IQR)**	0 (1)	2 (2.75)	0 (0)	0.2
**Surgery time (median/IQR)**	166.5 (84.7)	149 (121.5)	174 (79)	0.758
**Anesthesia time (median/IQR)**	93 (70.5)	92 (22.2)	93 (75.7)	0.956
**Insurance** (*n*/%)				
− Private/semi-private insurance	9 (18.7)	1 (8.3)	8 (22.2)	N/A
− General insurance	39 (81.2)	11 (91.6)	28 (77.7)	N/A
**Diagnosis** (*n*/%)				
− No Tumor	13 (27.0)	4 (33.3)	9 (25)	N/A
− Tumor	35 (72.9)	8 (66.6)	27 (75)	N/A
**Operation** (*n*/%)				
− Hemicolectomy left and Extended Hemicolectomy left	16 (33.3)	4 (33.3)	12 (33.3)	N/A
− Hemicolectomy right and Extended Hemicolectomy right	16 (33.3)	4 (33.3)	12 (33.33)	N/A
− Sigmoidectomy	16 (33.3)	4 (33.3)	12 (33.33)	N/A
**Anastomotic Leak Grading System (Rahbari Score)** (*n*/%)				
− A: Anastomotic Leakage results in no change	2 (4.2)	2 (16.7)	0 (0)	N/A
− B: Leakage requires intervention but no relaparatomy	2 (4.2)	2 (16.7)	0 (0)	N/A
− C: Leakage requires relaparatomy	8 (19)	8 (66.6)	0 (0)	N/A
**Anastomotic Technique** (*n*/%)				
− Stapler	5 (10.4)	5 (41.6)	0 (0)	N/A
− Hand-Sewn	26 (54.2)	6 (50)	20 (55.5)	N/A
− Stapler and Hand-Sewn	17 (35.4)	1 (8.4)	16 (44.4)	N/A

Significance: * *p* < 0.001. N/A: not applicable.

**Table 3:** Regression model—using the Anova function in R (dependent variable is Net Revenue).

Variable	Estimate	Std. Error	t Values	*p* Value	2.5% CI	97.5% CI
Insurance	10,453.19	4567.77	2.28	0.143	1045.70	19,860.69
Age	−159.61	227.70	−0.70	0.48	−628.56	309.34
Sex	5848.93	4212.52	1.38	0.17	−2826.92	14,524.79
Diagnosis	2088.05	3910.99	0.53	0.59	−5966.78	10,142.88
Operation	−1126.87	5141.15	−0.21	0.53	−11,715.27	9461.52
Approach	−4686.61	4745.13	−0.98	0.33	−14,459.38	5086.16
Hospitalization	−581.59	297.43	−1.95	0.06 *	−1194.15	30.97
Intensive Care time	−654.74	281.39	−2.32	0.02 *	−1234.27	−75.21
Surgical operating time	38.63	27.45	1.40	0.17	−17.90	95.17
Anesthesia Time	−31.10	38.17	−0.81	0.42	−109.70	47.51
Anastomotic Leakage	−11,416.60	7613.99	−1.49	0.01 *	−27,097.92	4264.69
Suture Stapler	−45,898.00	20,868.00	−2.19	0.05	−93,104.82	1309.22
Suture Hand-Sewn	13,895.00	28,256.00	0.49	0.63	−50,023.48	77,813.74
Rahbari Score A	−83,216.00	24,485.00	−1.92	0.10	−137,653.34	−17,777.66
Rahbari Score B	70,198.00	40,284.00	1.74	0.11	−20,930.87	161,326.87
Rahbari Score C	50,983.00	31,847.00	1.60	0.14	−21,061.07	123,026.32

Significance: * *p* < 0.05.

## Data Availability

The datasets used and/or analyzed during the current study are available from the corresponding author on reasonable request.

## Abbreviations

AL	Anastomotic Leak
CHF	Swiss Franc
ERAS	Enhanced Recovery After Surgery
IBD	inflammatory bowel disease
NHS	national healthcare system
PROM	Patient Reported Outcome Measures
PSM	Propensity Score Matching
SwissDRG	Swiss Diagnose Related Groups

## References

[1] Artus A., Tabchouri N., Iskander O., Michot N., Muller O., Giger-Pabst U., Bourlier P., Bourbao-Tournois C., Kraemer-Bucur A., Lecomte T. (2020). Long term outcome of anastomotic leakage in patients undergoing low anterior resection for rectal cancer. BMC Cancer.

[2] Matthiessen P., Hallböök O., Rutegard J., Simert G., Sjödahl R. (2007). Defunctioning stoma reduces symptomatic anastomotic leakage after low anterior resection of the rectum for cancer: A randomized multicenter trial. Ann. Surg..

[3] Yeh C.Y., Changchien C.R., Wang J.-Y., Chen J.-S., Chen H.H., Chiang J.-M., Tang R. (2005). Pelvic drainage and other risk factors for leakage after elective anterior resection in rectal cancer patients: A prospective study of 978 patients. Ann. Surg..

[4] Peeters K.C.M.J., Tollenaar R.A.E.M., Marijnen C.A.M., Klein Kranenbarg E., Steup W.H., Wiggers T., Rutten H.J., van de Velde C.J.H. (2005). Risk factors for anasto-motic failure after total mesorectal excision of rectal cancer. J. Br. Surg..

[5] Wallace B., Schuepbach F., Gaukel S., Marwan A.I., Staerkle R.F., Vuille-Dit-Bille R.N. (2020). Evidence according to Cochrane Systematic Reviews on Alterable Risk Factors for Anastomotic Leakage in Colorectal Surgery. Gastroenterol. Res. Pract..

[6] Kube R., Mroczkowski P., Granowski D., Benedix F., Sahm M., Schmidt U., Gastinger I., Lippert H., Study group Qualitätssicherung Kolon/Rektum-Karzinome (Primärtumor) (Quality assurance in primary colorectal carcinoma) (2010). Anastomotic leakage after colon cancer surgery: A predictor of significant morbidity and hospital mortality, and diminished tumourfree survival. Eur. J. Surg. Oncol..

[7] La Regina D., Di Giuseppe M., Lucchelli M., Saporito A., Boni L., Efthymiou C., Cafarotti S., Marengo M., Mongelli F. (2018). Financial Impact of Anastomotic Leakage in Colorectal Surgery. J. Gastrointest. Surg..

[8] Hammond J., Lim S., Wan Y., Gao X., Patkar A. (2014). The Burden of Gastrointestinal Anastomotic Leaks: An Evaluation of Clinical and Economic Outcomes. J. Gastrointest. Surg..

[9] Lee S.W., Gregory D., Cool C.L. (2019). Clinical and economic burden of colorectal and bariatric anastomotic leaks. Surg. Endosc..

[10] Turrentine F.E., Denlinger C.E., Simpson V.B., Garwood R.A., Guerlain S., Agrawal A., Friel C.M., LaPar D.J., Stukenborg G.J., Jones R.S. (2015). Morbidity, Mortality, Cost, and Survival Estimates of Gastrointestinal Anastomotic Leaks. J. Am. Coll. Surg..

[11] Angerer A. (2020). Lean-Exzellenz im OP-Management.

[12] Dekier L. (2012). The Origins and Evolution of Lean Management System. J. Int. Stud..

[13] Team R.C. R: A Language and Environment for Statistical Computing. https://www.gbif.org/tool/81287/r-a-language-and-environment-for-statistical-computing.

[14] Hyman N., Manchester T.L., Osler T., Burns B., Cataldo P.A. (2007). Anastomotic leaks after intestinal anastomosis: It’s later than you think. Ann. Surg..

[15] Alves A., Panis Y., Trancart D., Regimbeau J.M., Pocard M., Valleur P. (2002). Factors associated with clinically significant anastomotic leakage after large bowel resection: Multivariate analysis of 707 patients. World J. Surg..

[16] Slieker J.C., Daams F., Mulder I.M., Jeekel J., Lange J.F. (2003). Systematic review of the technique of colorectal anastomosis. JAMA Surg..

[17] Ashraf S.Q., Burns E.M., Jani A., Altman S., Young J.D., Cunningham C., Faiz O., Mortensen N.J. (2013). The economic impact of anastomotic leakage after anterior resections in English NHS hospitals: Are we adequately remunerating them?. Colorectal Dis..

[18] Agzarian J., Visscher S.L., Knight A.W., Allen M.S., Cassivi S.D., Nichols F.C., Shen K.R., Wigle D., Blackmon S.H. (2019). The cost burden of clinically significant esophageal anastomotic leaks-a steep price to pay. J. Thorac. Cardiovasc. Surg..

[19] SwissDRG AG Fallpauschalen in Schweizer Spitälern/Seite Was ist SwissDRG?. https://www.swissdrg.org/application/files/5115/0234/7269/170810_SwissDRG_Broschuere.pdf.

[20] Lerch M.M., Rathmayer M., Siegmund B., Wilke M., Wedemeyer H., Stallmach A., Mayerle J., Lammert F. (2020). Die Grenzen des G-DRG-Systems bei der Abbildung von Komplexität in der Universitätsmedizin [Limits of the G-DRG system to reflect Complexity in German University Hospitals]. Z. Gastroenterol..

[21] Angehrn P., Magunia P., Benjamin R. (2014). Fitnesskur für Schweizer Spitäler: Wege zu Besseren, Effizien-Teren und Profitableren Kliniken.

[22] Coopers P. Schweizer Spitäler: So Gesund Waren Die Finanzen 2019/Seite Ziel EBDITAR-Marge Entfernt Sich Weiter. https://www.pwc.ch/de/publications/2021/studie-schweizer-spitaeler-2019.pdf.

[23] Ljungqvist O., Scott M., Fearon K.C. (2017). Enhanced Recovery After Surgery. JAMA Surg..

[24] Van Rooijen S., Carli F., Dalton S., Thomas G., Bojesen R., Le Guen M., Barizien N., Awasthi R., Minnella E., Beijer S. (2019). Multimodal prehabilitation in colorectal cancer patients to improve functional capacity and reduce postoperative complications: The first international randomized controlled trial for multimodal prehabilitation. BMC Cancer.

[25] Carli F., Bousquet-Dion G., Awasthi R., Elsherbini N., Liberman S., Boutros M., Stein B., Charlebois P., Ghitulescu G., Morin N. (2020). Effect of Multimodal Prehabilitation vs. Postoperative Rehabilitation on 30-Day Postoperative Complications for Frail Patients Undergoing Resection of Colorectal Cancer. JAMA Surg..

[26] Berkel A.E.M., Bongers B.C., Kotte H., Weltevreden P., De Jongh F.H.C., Eijsvogel M.M.M., Wymenga A.N.M., Bigirwamungu-Bargeman M., Van Der Palen J., Van Det M.J. (2022). Effects of Community-based Exercise Prehabilitation for Patients Scheduled for Colorectal Surgery with High Risk for Postoperative Complications. Ann. Surg..

[27] Schuld J., Richter S., Folz J., Jacob P., Gräber S., Schilling M. (2008). Einfluss IT-gestützter klinischer Behandlungspfade auf die Patientenzufriedenheit an einer chirurgischen Universitätsklinik. DMW Dtsch. Med. Wochenschr..

